# Apoplastic sugar may be lost from grape berries and retrieved in pedicels

**DOI:** 10.1093/plphys/kiac262

**Published:** 2022-06-01

**Authors:** Yun Zhang, Ben-Min Chang, Berenice Burdet, Zhanwu Dai, Serge Delrot, Markus Keller

**Affiliations:** Department of Horticulture, Irrigated Agriculture Research and Extension Center, Washington State University, Prosser, WA, USA; Ste. Michelle Wine Estates, Prosser, WA, USA; Department of Horticulture, Irrigated Agriculture Research and Extension Center, Washington State University, Prosser, WA, USA; Department of Horticulture, Irrigated Agriculture Research and Extension Center, Washington State University, Prosser, WA, USA; INRAE, University of Bordeaux, ISVV, Villenave d’Ornon, France; Institute of Botany, Chinese Academy of Sciences, Beijing, China; INRAE, University of Bordeaux, ISVV, Villenave d’Ornon, France; Department of Horticulture, Irrigated Agriculture Research and Extension Center, Washington State University, Prosser, WA, USA

## Abstract

In ripening grape (*Vitis* sp.) berries, the combination of rapid sugar import, apoplastic phloem unloading, and water discharge via the xylem creates a potential risk for apoplastic sugar to be lost from the berries. We investigated the likelihood of such sugar loss and a possible sugar retrieval mechanism in the pedicels of different *Vitis* genotypes. Infusion of D-glucose-1-^13^C or L-glucose-1-^13^C to the stylar end of attached berries demonstrated that both sugars can be leached from the berries, but only the nontransport sugar L-glucose moved beyond the pedicels. No ^13^C enrichment was found in peduncles and leaves. Genes encoding 10 sugar transporters were expressed in the pedicels throughout grape ripening. Using an immunofluorescence technique, we localized the sucrose transporter SUC27 to pedicel xylem parenchyma cells. These results indicate that pedicels possess the molecular machinery for sugar retrieval from the apoplast. Plasmodesmata were observed between vascular parenchyma cells in pedicels, and movement of the symplastically mobile dye carboxyfluorescein demonstrated that the symplastic connection is physiologically functional. Taken together, the chemical, molecular, and anatomical evidence gathered here supports the idea that some apoplastic sugar can be leached from grape berries and is effectively retrieved in a two-step process in the pedicels. First, sugar transporters may actively retrieve leached sugar from the xylem. Second, retrieved sugar may move symplastically to the pedicel parenchyma for local use or storage, or to the phloem for recycling back to the berry.

## Teaser

Grape berry pedicels may retrieve sugar that is lost via the xylem following apoplastic phloem unloading in the berries.

## Introduction

The development and metabolism of a plant’s sink organs are supported by sugar unloaded from the phloem following long distance transport from source organs. The unloading pathway may switch from symplastic to apoplastic in sinks that accumulate osmotica to high concentrations (Patrick, 1997; Milne et al., 2018). In addition, sugar may diffuse out from the phloem during long-distance transport (van Bel, 1990). High concentrations of apoplastic sugars have been found in organs of different plant species, including bean (*Phaseolus vulgaris*) stems (Minchin and Thorpe, 1984), sugarcane (*Saccharum officinarum*) stems (Welbaum et al., 1992; Dong et al., 1994), tomato (*Solanum lycopersicum*) fruits (Ruan et al., 1996), and grape (*Vitis* sp.) berries (Wada et al., 2008; Keller and Shrestha, 2014). Water follows the unloaded sugar osmotically and may accumulate in growing sink tissues, transpire from them, or move back to the xylem for efflux (Münch, 1930). Xylem water efflux (backflow) has been observed in grape (Keller et al., 2006; Tilbrook and Tyerman, 2009; Zhang and Keller, 2017), kiwifruit (*Actinidia chinensis*) (Clearwater et al., 2009), tomato (Windt et al, 2009), soybean (*Glycine max*) (Bennett et al., 1984), and cowpea (*Vigna unguiculata*) (Pate et al., 1985). Consequently, sugars unloaded to the apoplast are at risk of moving passively into the xylem and being swept away or leached from the sink (Keller et al., 2006, 2015), which poses a potential challenge of losing valuable assimilates through xylem backflow.

Plants have evolved strategies to limit sugar loss through the xylem. For example, sugarcane internodes can store massive amounts of sucrose (up to 0.7 M) in both storage parenchyma cells and their surrounding apoplast (Welbaum and Meinzer, 1990). Specialized sclerenchyma cells function as an apoplastic barrier to prevent most solute movement to the adjacent xylem vessels (Welbaum et al., 1992; Walsh et al., 2005). Similarly, developing seeds are apoplastically isolated from their parent plants (Patrick and Offler, 2001). However, neither of these mechanisms applies to grape berries. Xylem vessels in ripening berries remain intact and capable of conducting water in either direction (Bondada et al., 2005; Keller et al., 2006, 2015; Chatelet et al., 2008; Choat et al., 2009; Zhang and Keller, 2017). Moreover, no apoplastic barrier was found in the berries (Chatelet et al., 2008), and apoplastic dyes infused at the stylar end readily move out of the berries (Keller et al., 2006; Tilbrook and Tyerman, 2009; Zhang and Keller, 2017). We hypothesize that grape berries employ a different strategy to limit the loss of apoplastic sugar after unloading from the phloem: in addition to rapid and active transport into the fruit vacuoles (Sarry et al., 2004; Vignault et al., 2005), sugar that is swept out of a berry by xylem backflow may be retrieved in the pedicel. Retrieval of sugar from xylem vessels back to the phloem has been studied in the collection phloem and along the transport phloem of diverse species (Fritz et al., 1983; Minchin and Thorpe, 1987; van Bel, 2003; Thorpe et al., 2005; Decourteix et al., 2006; Botha et al., 2008). However, it is unknown whether such a sugar retrieval mechanism also exists near sinks that accumulate high amounts of hexoses (e.g. up to 1.5 M in grape berries; Keller and Shrestha, 2014).

Retrieving apoplastic sugars back to the phloem requires active transport (van Bel, 2003; Ayre, 2011). In rice (*Oryza sativa*) leaves, xylem parenchyma cell (XPC) membranes abutting vessel pit membranes may be sites of active transport from the apoplast to the symplast (Botha et al., 2008). Patrick (1997) proposed that hexose transporters (HTs) may retrieve leached sugars from the root apoplast. In mature grape leaves, one presumed role of HT1, HT3, and HT5 is to retrieve hexoses that have been leached to the apoplast back into the phloem (Hayes et al., 2007). In addition, sucrose transporters (SUTs or SUCs) have been proposed as candidates for retrieval of apoplastic sucrose in petioles and stems (Stadler et al., 1995). SUT1 was immunolocalized to XPCs in stems of walnut (*Juglans regia*) and in leaves and stems of tobacco (*Nicotiana tabacum*), tomato, and potato (*Solanum tuberosum*), suggesting its possible role in removing sucrose from xylem vessels (Decourteix et al., 2006; Schmitt et al., 2008). Three SUCs and six HTs were identified in grape berries, and seven HTs were expressed in grape suspension cells (Davies et al., 1999; Fillion et al., 1999; Conde et al., 2006; Hayes et al., 2007; Lecourieux et al., 2014). Among these, SUC11 and SUC12 are high-affinity, low-capacity sucrose/H^+^ symporters, SUC27 is a low-affinity, high-capacity sucrose/H^+^ symporter that also transports glucose, fructose, and mannose, and HT1‒7 are H^+^-dependent HTs; their putative functions and locations in grapes, and phylogenetic trees were reviewed elsewhere (Afoufa-Bastien et al., 2010; Davies et al., 2012; Lecourieux et al., 2014; Walker et al., 2021). Although the functions and (sub-)cellular localization of these transporters have not been fully elucidated, they may move apoplastic sugar that has been unloaded from the phloem into mesocarp cells for vacuolar storage (Sarry et al., 2004; Vignault et al., 2005; Davies et al., 2012; Lecourieux et al., 2014; Savoi et al., 2021). However, it is unknown whether any of these sugar transporters are expressed in pedicels.

Following our hypothesis, after apoplastic sugars have been retrieved into the symplast, they may move back to the sieve elements (SEs) through a symplastic pathway (Fritz et al., 1983). The fluorescent probe 5,6-carboxyfluorescein (CF) has been extensively used as a symplastic tracer because its movement pattern is similar to that of sucrose (Grignon et al., 1989; Oparka and Read, 1992; Hernández-Hernández et al., 2020). Its nonfluorescent form, 5,6-CF diacetate (CFDA), is nonpolar and moves across cell walls and membranes. Once CFDA diffuses into a viable cell, esterases cleave it to the polar CF that can only move via plasmodesmata (PD) within the symplast (Wright et al., 1996; Botha, 2005). Using this tracer, Botha et al. (2008) demonstrated the symplastic pathway from XPCs to SEs in rice leaf blades. Whether such a symplastic pathway exists in fruit pedicels is unknown.

We carried out a series of experiments to test the hypothesis that some apoplastic sugar is leached from ripening grape berries and retrieved in their pedicels. First, we tested the possibility of sugar moving out of the berries by infusing ^13^C-labeled glucose, using both D-glucose-1-^13^C, which is recognized by HTs, and L-glucose-1-^13^C, which is not. Second, we ascertained the existence of the relevant molecular machinery in the pedicels by analyzing the expression of three *SUCs* (*SUC11*, *SUC12*, and *SUC27*), and seven *HTs* (*HT1‒7*). Next, we localized the most highly expressed sugar transporter, SUC27, in the pedicel, using an immuno-fluorescence technique. Finally, we examined the hypothesized symplastic connection between XPCs and phloem, using CF and transmission electron microscopy (TEM). Here we present chemical, molecular, and anatomical evidence to demonstrate that sugar can be leached from grape berries and retrieved in pedicels.

## Results

### Glucose-1-^13^C can move back from grape berries to pedicels

**Figure 1:**
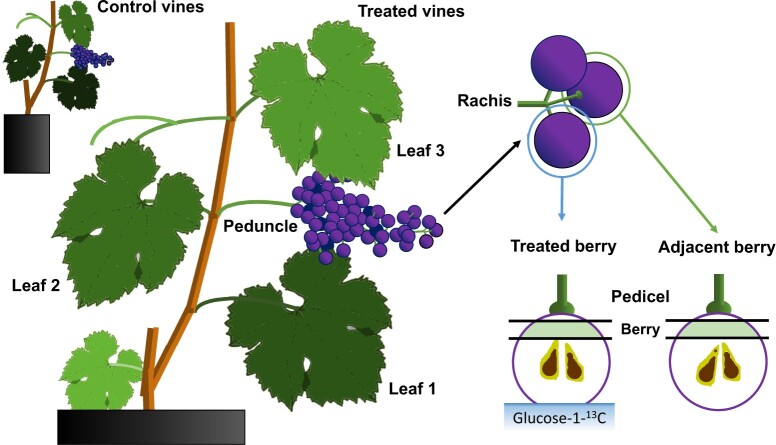
Illustration of glucose-1-^13^C reverse infusion and sampling of grapevine organs. The cut stylar end of a treated berry was immersed in glucose-1-^13^C solution. Samples collected after 3 h or 24 h of infusion included the proximal ends of treated and adjacent berries, their pedicels, rachis, peduncle, and leaves 1, 2, and 3. Control samples were taken from untreated vines or vines treated with nonlabeled glucose solution.

**Figure 2:**
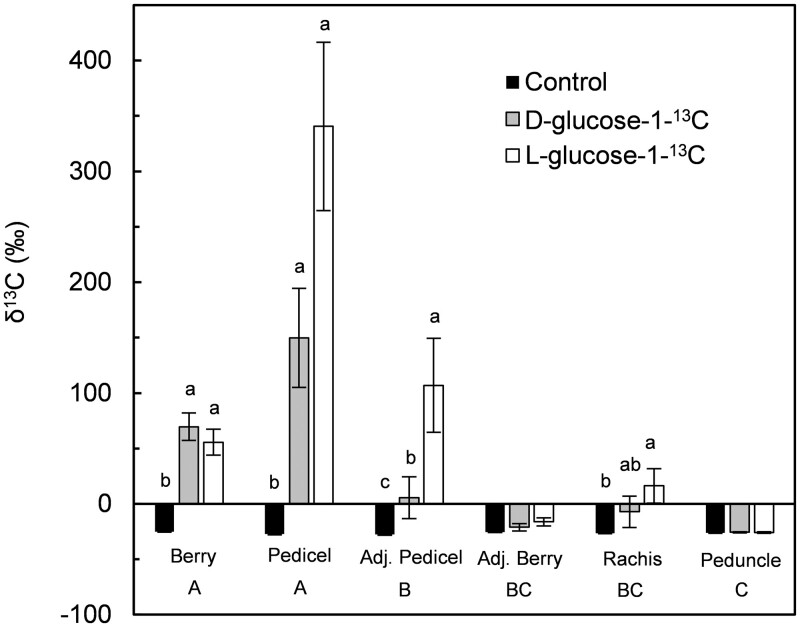
Carbon isotope ratio (δ^13^C) in various organs of Merlot grapevines following infusion of D-glucose-1-^13^C (transport sugar) or L-glucose-1-^13^C (nontransport sugar) at the stylar end of grape berries for 24 h. Control values represent the natural δ^13^C abundance in organs of untreated vines. Samples from the same cluster included berries (cross section downstream from feeding site) and their pedicles, as well as berries (Adj. berry) and pedicels (Adj. pedicel) immediately adjacent to treated berries, and the subtending cluster rachis and peduncle. Values are means ± se (*n* ≥4 individual organs). Uppercase letters indicate significant differences among tissues, and lowercase letters indicate significant differences between the control and the two feeding sugars, at *P* < 0.05 by Tukey’s honest sigificant difference test of log-transformed data.

We tested the possibility of sugar being leached from ripening grape berries by infusing either D-glucose-1-^13^C (transport sugar) or L-glucose-1-^13^C (nontransport sugar; Oliveira et al., 2002) at the stylar end of Concord and Merlot berries (Figure 1). We used glucose, rather than the phloem transport sugar sucrose, because sucrose is rapidly hydrolyzed in the apoplast of grape berries (Keller and Shrestha, 2014). The berries used in these experiments were at the early ripening stage (10‒15°Brix), when sugar import via the phloem is most rapid (Keller et al., 2015; Zhang and Keller, 2017; Zhu et al., 2019). Control organs (berries, pedicels, rachises, peduncles, leaves) collected from either untreated plants or plants whose berries were infused with unlabeled glucose had similar natural ^13^C abundance (mean ± standard error (se), of δ^13^C = −26.5 ± 0.2; *P* = 0.22). In both genotypes, glucose-1-^13^C feeding led to significantly higher δ^13^C than in the control (with or without nonlabeled glucose feeding) in berry tissues downstream from the feeding site (i.e. in the seeds‒receptacle region) and in subtending pedicels (Table 1 and Figure 2). Higher δ^13^C meant enrichment with ^13^C, indicating glucose-1-^13^C had moved back from the distal stylar end of a treated berry toward its proximal end and out to the pedicel. Merlot berries that were infused for 24 h in an independent experiment had a much higher level of enrichment (Figure 2) compared with berries infused for 3 h (Table 1). Even after 24 h of infusion, ^13^C enrichment in the rachis was only significant for L-glucose-1-^13^C but not for D-glucose-1-^13^C (Figure 2). Irrespective of the feeding sugar used, no enrichment was found in the cluster peduncle or in the adjacent leaves. Berries that were immediately adjacent to the treated berries also did not have any ^13^C enrichment compared with the control, whereas their pedicels showed stronger enrichment with L-glucose-1-^13^C than with D-glucose-1-^13^C (Figure 2). These results indicate that sugar can be leached from grape berries to pedicels but not beyond, unless the sugar is in a form not recognized by sugar transporters.

**Table 1:** Carbon isotope ratio (δ^13^C) in various organs of Concord and Merlot grapevines following glucose-1-^13^C feeding (^13^C feeding) to the stylar end of grape berries for 3 h

Genotype	Organ	Control δ^13^C (‰)	^13^C feeding δ^13^C (‰)	*P*
Concord	Berry	−25.9 ± 0.5	−10.9 ± 5.2	0.008
	Pedicel	−27.9 ± 0.4	−22.2 ± 1.9	0.02
	Adj. berry		−26.8 ± 0.8	0.69
	Adj. pedicel		−27.3 ± 0.3	0.16
	Rachis	−26.5 ± 0.5	−26.4 ± 0.3	0.84
	Peduncle	−26.3 ± 0.2	−26.7 ± 0.2	0.23
Merlot	Berry	−25.1 ± 0.4	−21.2 ± 0.7	<0.001
	Pedicel	−26.9 ± 0.3	−23.8 ± 1.3	0.04
	Adj. berry		−24.4 ± 0.4	0.10
	Adj. pedicel		−26.5 ± 0.1	0.28
	Rachis	−25.7 ± 0.2	−23.7 ± 1.7	0.37
	Peduncle	−25.3 ± 0.3	−25.0 ± 0.1	0.57

Control values represent the natural δ^13^C abundance in organs sampled from untreated vines. Samples from the same cluster included berries (cross sections downstream from feeding sites) and their pedicles, as well as berries (Adj. berry) and pedicels (Adj. pedicel) immediately adjacent to treated berries, and the subtending cluster rachis and peduncle. Values are means ± se (*n* ≥3 individual organs). The δ13C data were tested by Bartlett’s test for homogeneity of variances and log-transformed. Effects of 13C feeding by organ and genotype were analyzed by *t*-test.

### Sugar transporter genes are expressed in pedicels

**Figure 3:**
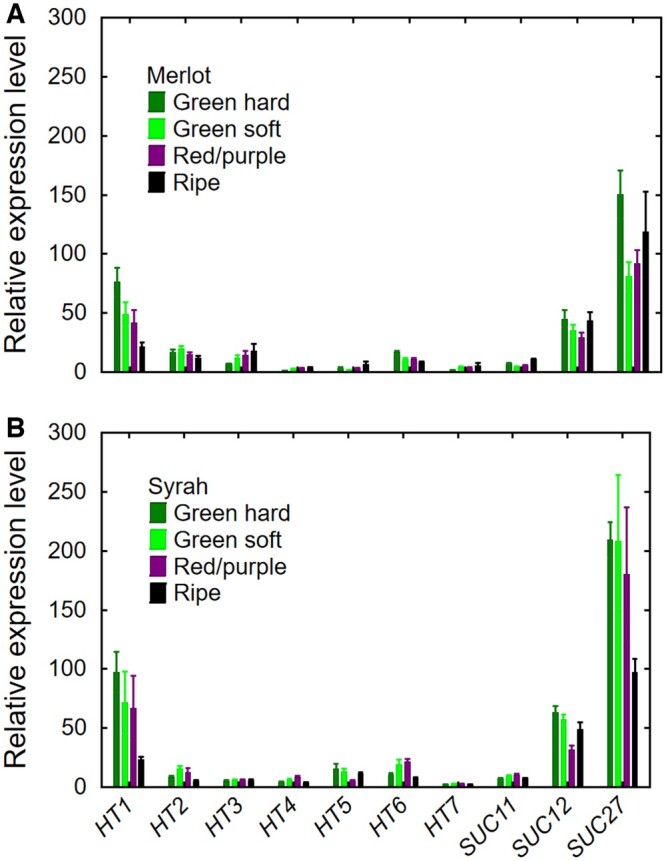
Relative expression of 10 sugar transporter genes (*HT1, HT2, HT3, HT4, HT5, HT6, HT7, SUC11, SUC12*, and *SUC27*) in grape berry pedicels at four developmental stages (green hard, green soft, red/purple, and ripe). A, Merlot. B, Syrah. Values are means ± SE (*n* = 6; 50 pedicels per replicate).

To test the expression of sugar transporter genes in grape berry pedicels, we initially grouped Merlot and Syrah berries into the developmental stages green hard, green soft, red/purple, and ripe according to Zhang and Keller (2015, 2017) and Hernández-Montes et al. (2021). All seven *HTs* and three *SUCs* were expressed in the pedicels at all developmental stages (Figure 3). Expression levels did not differ between genotypes (*P* = 0.26). In both genotypes, the most-expressed gene was *SUC27*, followed by *HT1* and *SUC12* (three-fold lower); the other seven genes were expressed at similar and lower levels (*P* < 0.05). In both genotypes, *HT1* expression was highest when the berries were green hard and decreased at ripeness (*P* < 0.05), while the developmental stage did not alter the expression of *HT3*, *HT5*, *HT7*, and *SUC27* (*P* > 0.10). In Merlot, *HT2* and *SUC12* expression was similar across developmental stages (*P* ≥ 0.10), but *HT4* expression increased and that of *HT6* decreased during ripening (*P* < 0.01) (Figure 3A). In Syrah, the developmental patterns of *HT2*, *HT4*, and *HT6* exhibited similar bell curves with a peak at the green soft or red/purple stage (*P* < 0.01), while *SUC12* expression was lower at the red/purple stage (*P* < 0.01) (Figure 3B).

**Figure 4:**
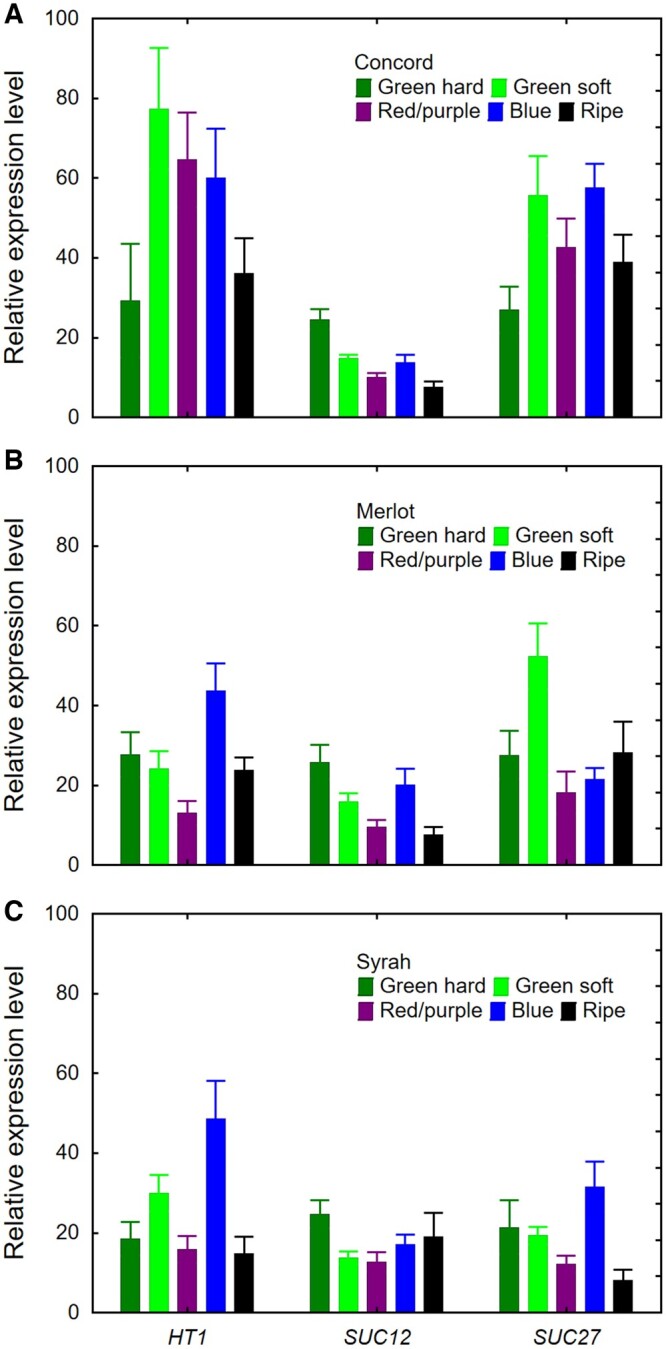
Relative expression of three sugar transporter genes (*HT1, SUC12*, and *SUC27*) in grape berry pedicels at five developmental stages (green hard, green soft, red/purple, blue, and ripe). A, Concord. B, Merlot. C, Syrah. Values are means ± SE (*n* = 6; 20 pedicels per replicate).

We next focused on the three most highly expressed genes (*HT1*, *SUC12*, and *SUC27*) and included one additional grape genotype (Concord, a hybrid grape with *Vitis labrusca* and *Vitis vinifera* ancestry) and one additional developmental stage (blue). All three genes were expressed in the pedicels of all three genotypes (Figure 4). Except for *SUC12*, gene expression was higher in Concord than in Merlot or Syrah (*P* < 0.001). Concord berries are about twice the size of the berries of the two *V.vinifera* cultivars and accumulate much more sugar (Chang et al., 2019). In Concord, *HT1* expression was similar across developmental stages (*P* = 0.07); *SUC12* expression decreased during ripening (*P* < 0.001); *SUC27* expression was lower at the green hard stage than at the green soft and blue stages (*P* < 0.05) (Figure 4A). Different from our earlier results, *HT1* expression was highest at the blue stage of both Merlot and Syrah (*P* < 0.001); the expression level of *SUC12* was highest at the green hard stage for both genotypes (*P* < 0.05); and *SUC27* expression was highest at the green soft stage in Merlot and at the blue stage in Syrah (*P* < 0.01) (Figure 4, B and C).

### The SUT SUC27 is localized in pedicel XPCs

**Figure 5:**
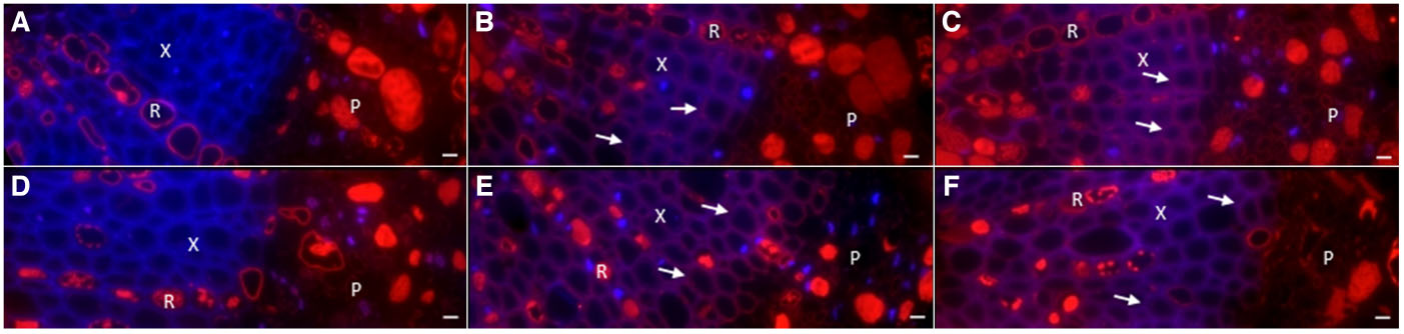
Localization of SUC27 in XPCs of grape berry pedicels. A‒C, Merlot. D‒F, Concord. In the negative controls (A and D) cell nuclei and lignified cell walls appear blue (stained with DAPI); red autofluorescence is due to phenolic compounds. In green hard (B and E) and ripe (C and F) stages SUC27 appears purple due to red fluorescence overlapping with blue fluorescence (arrows) (stained with αSUC27 antibody and labeled with Texas Red). P, phloem; R, ray; X, xylem. Scale bar = 20 µm.

Using an αSUC27 antibody we localized SUC27 to pedicel XPCs. Figure 5 shows the results for green hard and ripe berries of Merlot and Concord; results were similar for Syrah and for the other developmental stages tested (green soft, red/purple, and blue). The SUC27 protein appeared as purple due to overlapping red and blue fluorescence (arrows in Figure 5, B, C, E, and F). No overlapping fluorescence was detected in the negative controls using the same procedure without the primary antibody (Figure 5, A and D). The blue fluorescence was due to 4′,6-diamidino-2-phenylindole (DAPI) labeling cell nuclei and lignified cell walls in the xylem and phloem. XPCs in grape berry pedicels are heavily lignified (Knipfer et al., 2015). Red fluorescence in rays and phloem cells was due to autofluorescence of phenolic compounds and, like the blue fluorescence, occurred in both controls and antibody-treated samples. Attempts to localize SUC12 and HT1 were unsuccessful, which may be due to low levels of proteins, poor access of antibodies to proteins, and/or design of the antibodies.

### Plasmodesmata connect vascular parenchyma cells

Using light microscopy, we observed the same anatomical features in the pedicel cross-sections of Concord, Merlot, and Syrah berries examined at green hard, green soft, and ripe stages. Supplemental Figure S1 shows a cross section of a Merlot pedicel at the green hard stage. The xylem was composed of vessels with secondary cell walls and XPCs. The cambium comprised a few layers of nearly rectangular cells abutting the xylem and phloem regions. Cells in the phloem were generally smaller and more compact than elsewhere. Cells in the cortex were much larger than those in the vascular tissues. Moreover, cortical cells were loosely arranged with apparent intercellular spaces. The dark material in the parenchyma cells was likely to be phenolic compounds.

**Figure 6:**
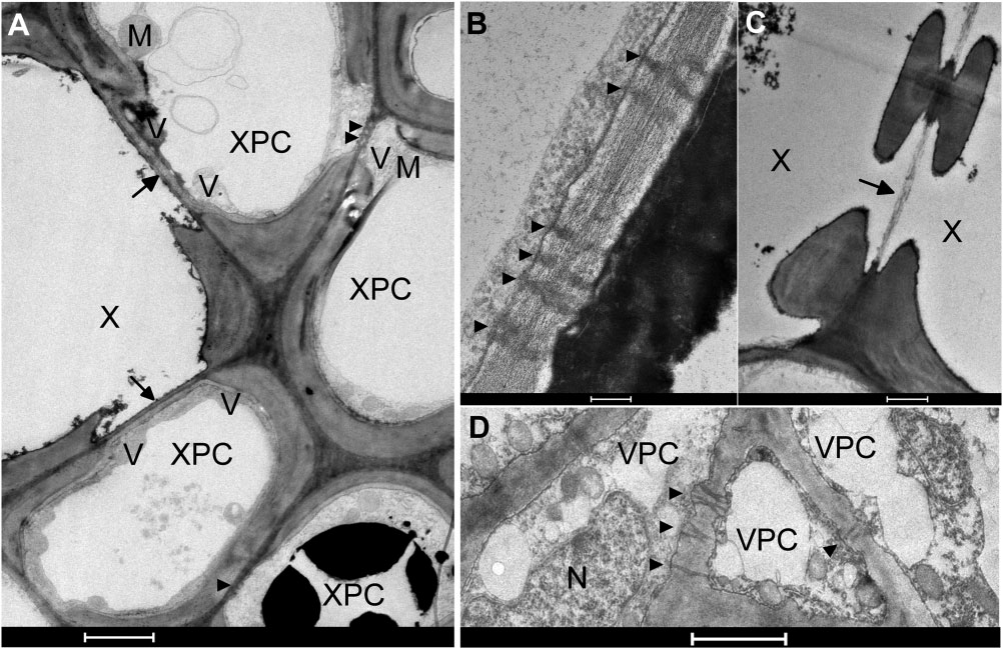
Ultrastructure of the vascular tissues in pedicel cross sections of grape berries. A, Xylem vessels (X) and XPCs. Arrow indicates hydrolyzed vessel cell wall abutting an XPC. Arrow heads indicate plasmodesmata between XPCs. B, Close-up of plasmodesmata (arrow heads) between XPCs. C, Two vessels with bordered pits and hydrolyzed vessel cell wall (arrow). D, VPC interconnected by plasmodesmata (arrow heads). M, mitochondrion; N, nucleus; V, vesicle; X, xylem vessel. Bars = 2 μm in A and D; bar = 0.2 μm in (B); bar = 1 μm in (C).

The ultrastructure of the vascular tissues in Concord, Merlot, and Syrah pedicel cross sections was observed under TEM (Figure 6). Xylem vessels displayed the typical features of thickened cell walls and bordered pits with pit membranes on the vessel side. Vesicles were often observed in the XPCs abutting vessels. The XPCs and vascular parenchyma cells (VPCs) were connected via PD. Most PD appeared normal, that is, not blocked by electron-opaque material (van Bel, 2003), and exhibited simple structure; some PD appeared branched at one or both ends (Figure 6B). Organelles, such as mitochondria, chloroplasts, and nuclei, were observed in both XPCs and VPCs.

### Carboxyfluorescein moves symplastically in pedicel vascular tissues

**Figure 7:**
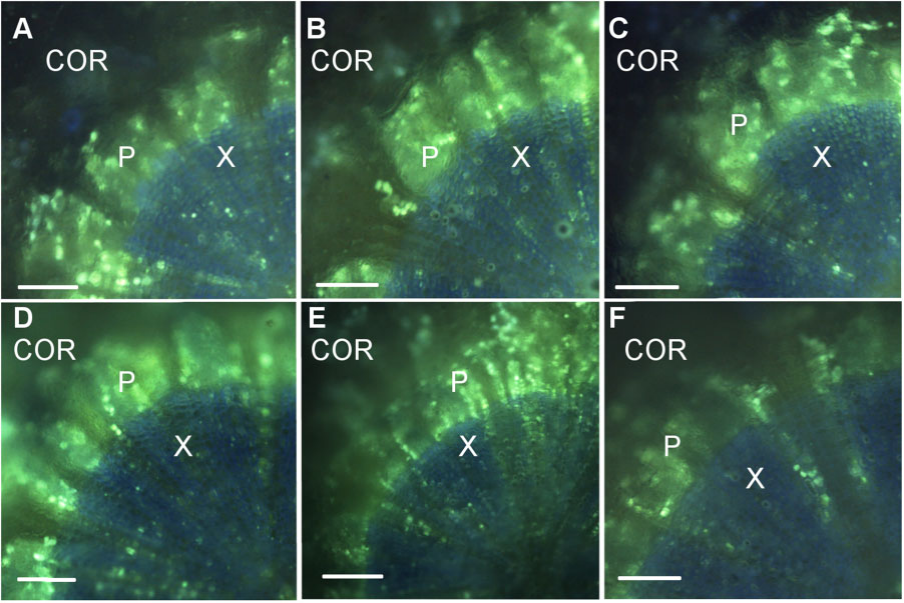
Grape berry pedicel cross sections at three developmental stages. A‒C, Syrah. D‒F, Concord. Stages included green hard (A and D), green soft (B and E), and ripe (C and F). Images were captured 24 h after feeding 5,6-CFDA through cut peduncles. Bright green 5,6-CF fluorescence was detected in the phloem (P) and xylem (X) but not in the cortex (COR). Dull blue autofluorescence in the xylem is due to lignified cell walls. Bars = 100 μm.

We confirmed the TEM results by using the symplast-mobile dye CF to verify the existence of functional symplastic connections in the pedicels. During infusion through the cut peduncle base of Concord and Syrah clusters, CFDA would initially have been transported in xylem vessels (remaining nonfluorescent) and gradually diffused into adjacent XPCs, where it was cleaved to the fluorescent CF (Wright and Oparka, 1996). In the pedicel cross sections examined ≥10 cm away from the cut end of the peduncle, we observed bright green fluorescence in the xylem and phloem regions, but much more concentrated in the latter (Figure 7). The blue fluorescence in the xylem, which partly masked the green fluorescence, was caused by autofluorescence of lignin in vessel cell walls under the ultraviolet light (Hutzler et al., 1998; Knipfer et al., 2015), confirming the heavy lignification across the xylem observed in Figures 5 and 6. Little green fluorescence was observed in the cortex (Figure 7). The distribution pattern of green fluorescence from CF was consistent in both genotypes and across all tested developmental stages from green hard to ripe.

## Discussion

This study demonstrated that small amounts of sugar can be leached back to the pedicel from fleshy fruit, such as grape berries, that employ apoplastic phloem unloading. It also provided several lines of evidence in support of the existence of a sugar retrieval mechanism in pedicels: (1) ^13^C-labeled glucose infused through a berry’s stylar end moved through the berry’s proximal end to the pedicel, and unlike D-glucose-1-^13^C, the nontransport sugar L-glucose-1-^13^C also moved to the subtending rachis, but neither sugar moved to the peduncle and adjacent leaves; (2) 10 sugar transporter genes were expressed in the pedicels of three grape genotypes, providing the molecular machinery to retrieve sugar from the apoplast back into the symplast; (3) SUC27 was localized to pedicel XPC in close proximity to vessels; (4) plasmodesmata were observed between parenchyma cells in the pedicel vascular tissues, permitting symplastic sugar movement after its retrieval from the apoplast; and (5) the distribution of CF confirmed that the symplastic connection in the pedicel vascular tissues is physiologically functional. Unlike xylem-mobile dye, which readily moved from grape berries to other parts of a treated cluster and back to the leaves within 3 h of reverse infusion (Keller et al., 2006), glucose-1-^13^C did not move beyond a treated berry’s pedicel and occasionally into an adjacent pedicel even after 24 h, unless the sugar was in a form not recognized by sugar transporters (Figure 2). These findings suggest that berries effectively remove most sugar from the apoplast and that the amount of leached apoplastic sugar is small and may be retrieved in the pedicels, preventing it from being swept away in the xylem. Thus, our data support the idea of a two-step sugar retrieval process in pedicels. In step 1, active trans-membrane transport in XPCs may move sugar from the apoplast to the symplast; in step 2, sugar may move symplastically to the surrounding VPCs or the phloem. Retrieved sugars may be utilized or stored locally in the pedicels and/or may be transported back to the berries via the phloem.

### Sugar retrieval step 1: trans-membrane transport from the apoplast to the symplast

We found that 10 genes encoding sugar transporters in *Vitis* were expressed in the pedicels of different genotypes at various developmental stages (Figures 3 and 4). This demonstrates that the molecular machinery required for the retrieval of sugar from the apoplast is available and functional at the transcription level. Although expression of these transporter genes *per se* says nothing about the direction of transport, sugar/H^+^ symport into the symplast should be facilitated by the acidic apoplast pH (Keller and Shrestha, 2014). There was no consistent developmental pattern in expression profiles among different transporters and genotypes, suggesting that sugar transporters are constitutively expressed in pedicels throughout berry development and/or are regulated at the posttranscriptional level. High variation in expression of the same genes analyzed in different grapevine organs have previously been reported (Davies et al., 1999; Fillion et al., 1999; Hayes et al., 2007; Afoufa-Bastien et al., 2010), but we found no prior information for pedicels. Under constitutive gene expression, sugar transporter activity may be regulated “on demand” by phosphorylation or protein turnover. For instance, transfer of *Arabidopsis thaliana* plants from low to high light increased photosynthesis, leaf and phloem sugar content, and SUC2 phosphorylation, and reduced SUC2 ubiquitination, without altering *SUC2* gene expression (Xu et al., 2020).

Among the seven *HT* genes evaluated in the pedicels, *HT1* had the highest expression. In grape berries, *HT1‒3* are much more highly expressed than the other *HT*s at all stages of berry development, but unlike *HT2* and *HT3*, *HT1* expression declines during berry development (Davies et al., 2012; Lecourieux et al., 2014; Ren et al., 2020). The HT1 protein was localized to the plasma membranes of both the sieve element/companion cell (SE/CC) complex and the mesocarp cells and was proposed to be involved in hexose retrieval in leaves, petioles, and berries (Vignault et al., 2005, Hayes et al., 2007). HT3‒5 are also located in the plasma membrane, whereas HT2 and HT6 are thought to be localized in the tonoplast and may be involved in vacuolar sugar accumulation during fruit ripening (Davies et al., 2012; Lecourieux et al., 2014). The cellular location and transport function of HT7 are unknown. Among the three sucrose/H^+^ symporters, whose (sub-)cellular locations are also unknown, *SUC11* and *SUC12* were upregulated, while *SUC27* was downregulated, in Syrah berries at the beginning of ripening (Davies et al., 1999; Lecourieux et al., 2014). Although we did not observe this developmental pattern in grape pedicels, *SUC27* was among the most highly expressed sugar transporters (Figures 3 and 4), and we demonstrated that SUC27 is located in pedicel XPC membranes (Figure 5). As a member of the SUC2/SUT1 subfamily, grapevine SUC27 is closely related to walnut SUT1 (Sauer, 2007); *SUT1* was expressed in XPCs of walnut trees and was proposed to recover sucrose from xylem vessels to the symplast (Decourteix et al., 2006).

Since genes encoding both hexose and SUTs were expressed in grape pedicels, the retrieved sugar may be in the form of hexoses and/or sucrose. Unlike in grape berries, comparable concentrations of hexoses and sucrose, as well as starch, were found in pedicels (Amerine and Root, 1960; Nii and Coombe, 1983); thus hexoses could be reassembled to sucrose in the symplast for reloading into the phloem, or converted to starch for local storage. In our ^13^C-labeled sugar infusion experiments, the nontransport sugar L-glucose, but not the transport sugar D-glucose, moved beyond the pedicels of infused and immediately adjacent berries, but neither sugar moved back to the peduncle within 24 h (Figure 2). Mitochondria and membrane-bound vesicles present in pedicels (Figure 6) also support the possibility of active transport from the apoplast. Thus, the first step of sugar retrieval is likely to be predominantly an active process. Retrieval by passive diffusion down a concentration gradient (Tomkins et al., 2021) may have occurred for L-glucose, but it was clearly less effective than the retrieval of D-glucose (Figure 2).

### Sugar retrieval step 2: plasmodesmata facilitate symplastic transport

We demonstrated that grape pedicel VPCs are symplastically connected via PD (Figure 6) and that this connection is physiologically functional (Figure 7). Simple PD, which were more frequently observed, provide a low-resistance transport pathway for solute movement through the symplast (Oparka et al., 1999). Because the existence of PD and their frequency alone do not guarantee the transport capacity or functionality of PD (Fisher and Oparka, 1996), we used CFDA feeding to demonstrate a functional symplastic connection in both the xylem and phloem regions of pedicels. Within symplastic fields, CF moves through PD by advection down turgor gradients and by diffusion down concentration gradients (Hernández-Hernández et al., 2020; Tomkins et al., 2021). By contrast, we observed little CF dye in the cortex (Figure 7), which indicates that the cortex has limited symplastic connections with the phloem region. A functional symplastic connection between pedicel VPCs provides a possible low-resistance pathway for the movement of retrieved sugar back to the phloem. Quantification of the concentration gradient required for such passive sugar movement is technically challenging, but published values of the sugar concentration in phloem sap from grape pedicels are ≤50 mM (Zhang et al., 2006; Zhang and Keller, 2017), whereas that in whole pedicels is roughly 100 mM (Amerine and Root, 1960; Nii and Coombe, 1983). Since the loosely packed cortex makes up nearly 80% of the pedicel cross section and the xylem comprises vessels in addition to XPCs (Figure 6; Supplemental Figure S1), XPCs and VPCs are likely to contain more than 100-mM sugar. The loose arrangement of the cortical cells, with prominent intercellular spaces, is typical of tissues involved in gas exchange with the atmosphere. Indeed, pedicels were recently demonstrated to play a major role in supplying oxygen to ripening grape berries (Xiao et al., 2018).

### Alternative strategy needed: apoplastic sugar unloading meets xylem backflow

During grape berry ripening, phloem unloading follows an apoplastic pathway (Zhang et al., 2006), and the concentration of apoplastic sugars increases to as much as 1.5 M along with sugar accumulation in the vacuoles (Wada et al., 2008, 2009; Keller and Shrestha, 2014). The small volume of the berry apoplast (Diakou and Carde, 2001) and the active retrieval of unloaded sugar into the symplast (Sarry et al., 2004) may limit apoplastic sugar leaching. However, considering that surplus phloem water may be discharged by xylem backflow from the berries (Keller et al., 2015; Zhang and Keller, 2017), some apoplastic sugar leaching through the xylem is plausible (Table 1). In the absence of an apoplastic barrier (Keller et al., 2006; Chatelet et al., 2008; Tilbrook and Tyerman, 2009) that would prevent such sugar leaching, an alternative strategy would be to retrieve leached sugar en route to minimize losses of precious assimilates. The evidence provided here suggests that in sink organs (e.g. fruits) with xylem backflow and high apoplastic sugar concentration, sugar may be leached through the xylem and be retrieved from the apoplast to the symplast. Sugar leaching from the phloem and retrieval back to the phloem or surrounding parenchyma cells is common along the transport phloem (Minchin and Thorpe, 1987; Tixier et al., 2018); thus, ripening grape berries may simply utilize the existing retrieval machinery to minimize loss of sugar.

**Figure 8:**
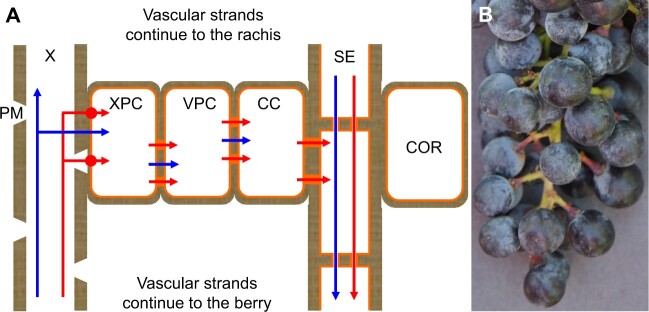
Conceptual model of proposed sugar retrieval mechanism in grape berry pedicels (not to scale). A, Some sugar that has been unloaded from the phloem to the berry apoplast is leached out (red arrow) in xylem vessels (X) along with xylem backflow (blue arrow in X). In the pedicel the sugar is actively transported back into XPCs by sugar transporters (filled circle) in the cell membranes (orange lines). Retrieved sugar may move symplastically through plasmodesmata (filled square) to vascular parenchyma cells, CC, and SEs for transport to the berry. The pedicel cortex (COR) is symplastically isolated from the phloem and facilitates gas exchange for respiration. B, Red pedicels in ripening grape cluster.

A conceptual model of our proposed sugar retrieval mechanism is shown in Figure 8A. After being unloaded from the release phloem to the berry apoplast, a small portion of the apoplastic sugar may move out of the berry along with xylem backflow (see also Keller et al., 2015). Unlike xylem water, however, the leached sugar rarely moves beyond the pedicel. Sugar transporters in the pedicel XPC membranes actively retrieve the sugar back to the symplast, and functional symplastic connections via PD facilitate sugar movement via VPCs back to the phloem. The loosely packed cortex that connects to numerous lenticels on the pedicel surface facilitates gas exchange with the atmosphere (see also Xiao et al., 2018), so that at least some of the energy required to fuel active transport could be provided by the retrieved sugar itself through respiration in the XPCs and VPCs. Retrieval of apoplastic sugars (and possibly other solutes such as potassium) that have been leached from the fruit could be physiologically important in conserving valuable assimilates and osmolytes transported to the sink. The retrieved sugar may be utilized for growth of the surrounding tissues (e.g. to match pedicel transport capacity to fruit sink demand), stored there (Minchin and Thorpe, 1987; Tixier et al., 2018), or recycled back to the ripening fruits via the phloem. Localized sugar accumulation would also explain the phenomenon of pedicels and subtending rachis sections sometimes turning red during grape ripening (Figure 8B); reddening of grape tissues generally indicates sugar-induced anthocyanin accumulation (Keller, 2020).

## Conclusions

Apoplastic phloem unloading and sugar accumulation in ripening grape berries are coupled to discharge of surplus phloem water via the xylem, which poses a risk of apoplastic sugar being swept away by xylem backflow. Here we provide evidence for such sugar leaching from berries back to their pedicels and for a two-step sugar retrieval mechanism in the pedicels. The expression of sugar transporter genes in the pedicels and the localization of one of these transporters in XPC provide the molecular machinery to retrieve sugar from the apoplast back into the symplast. Anatomical observations coupled with the movement of fluorescent dye indicate that the retrieved sugar may move symplastically through PD back to parenchyma cells and/or the phloem. This retrieval mechanism appears to be very effective, as we did not observe sugar leaching in grape clusters beyond the pedicels, unless the sugar was in a form not recognized by sugar transporters.

## Materials and methods

### Plant material

We used own-rooted grapevines *Vitis vinifera* cvs. Merlot and Syrah (planted in 1999) and *Vitis labruscana* cv. Concord (planted in 2003) for sample collection from 2012 through 2019. Grapevines were grown in experimental vineyards at the Irrigated Agriculture Research and Extension Center in Prosser, Washington, USA (46°17′ N; 119°44′ W; elevation 365 m). The vines were drip-irrigated and grown at a planting distance of 1.8 m (within rows) by 2.7 m (between rows) in north–south-oriented rows down a ≤ 2% south-facing slope.

Own-rooted Merlot and Concord grapevines propagated from dormant cuttings taken from the field-grown plants were grown in white 20-L pots containing a mixture of 50% sandy loam, 25% peat moss, 25% pumice, and 3 g L^−1^ dolomite. These vines were grown outside and moved into an air-conditioned greenhouse (temperature 18–25°C) for each experiment. Supplemental light maintained a minimum photoperiod of 12 h and midday photosynthetically active radiation >1000 μmol photons m^−2^ s^−1^. The pots were irrigated daily, and 5 g of Mora-Leaf Plus fertilizer (Wilbur–Ellis) was applied to each pot before anthesis.

### Reverse infusion of glucose-1-^13^C and carbon isotope analysis

In a first experiment, we fed aqueous solutions of D-glucose-1-^13^C (500 mM; 99 atom-% ^13^C, Omicron Biochemicals) to the stylar (distal) end of ripening grape berries on intact, 2-year-old pot-grown Merlot and Concord vines using the same approach as that used for reverse xylem-mobile dye infusion (Keller et al., 2006; Zhang and Keller, 2017). Briefly, well-watered vines were brought to full hydration using root pressure chambers. The stylar end of each treated berry was removed with a fresh razor blade, the cut end was immersed in D-glucose-1-^13^C solution, and the pressure on the roots was released. After 3 h, the treated berries (*n* = 8‒10 individual berries) and control (without infusion) berries (*n* = 6) with pedicels were removed from the vine. Additionally, a berry immediately adjacent to the treated berry and their shared cluster rachis (1-cm section, *n* = 3) and peduncle (3-cm section, *n* = 3) were sampled (Figure 1). For each berry, its pedicel (including most of the receptacle) and a 2-mm thick proximal berry cross section between the seeds and the receptacle end (i.e. downstream from the feeding site) were sampled. The remaining portion of the mesocarp was used to measure the total soluble solids by refractometry (Quick-Brix 60, Mettler-Toledo).

In a second experiment with 7-year-old potted Merlot grapevines, instead of using a root pressure chamber, we started glucose-1-^13^C reverse infusion when leaf guttation was observed before sunrise to ensure positive xylem pressure. In this experiment we used either D-glucose-1-^13^C or L-glucose-1-^13^C (500 mM; 99 atom-% ^13^C, Omicron Biochemicals) in addition to a nonlabeled D-glucose control and a blank control without glucose infusion and without stylar tip removal. After 24 h of infusion, we collected samples as described above; in addition to berry and cluster tissues, we also collected leaves (including leaf blades and petioles) at positions 1, 2, and 3 where leaf 2 was on the same node as and opposite from the treated cluster (Figure 1). The types and locations of sampled tissues were selected along the pathway of xylem backflow from berries to leaves (Keller et al., 2006). We collected at least three independent biological replicates (*n* = 3‒4 individual organs) of each (blank) control organ (berry, pedicel, rachis, peduncle, leaf) and at least seven replicates (*n* = 7‒8 individual organs) of treated berries, their pedicels, rachis, peduncle, and leaf. One peduncle sample from the D-glucose-1-^13^C treatment was discarded due to contamination during sample preparation.

Samples were wrapped individually in aluminum foil, oven-dried overnight at 60°C, and ground with a mortar and pestle. Ground samples (2 mg) were analyzed by continuous flow isotope ratio mass spectrometry at the Washington State University Stable Isotope Core Laboratory. Carbon isotope ratios were normalized using two running standards calibrated to internationally distributed isotopic reference materials (NBS 19, RM 8542, and IAEA-CO-9), and reported relative to Vienna Peedee belemnite (VPDB) as δ^13^C (‰) following Eq. 1:

Eq. 1 δ13C = ((C13 C12)sample(C13C12)VPDB−1) x1000‰

The δ^13^C data were tested by Bartlett’s test for homogeneity of variances and log-transformed. Effects of infusion treatments on δ^13^C for each organ of each genotype and infusion duration were analyzed by *t*-test in Microsoft Excel (version 14, Microsoft Corporation). Multiple range comparisons for sampling locations were conducted by Tukey’s HSD test in R (version 4.1.3, R Core Team).

### Ribonucleic acid extraction, complimentary deoxyribonucleic acid synthesis, and semiquantitative analysis of gene expression by reverse-transcription-quantitative polymerase chain reaction

We collected six biological replicates of 50 berries per replicate at each of four developmental stages (green hard, green soft, red/purple, ripe) from field-grown Merlot and Syrah vines. In a second experiment, we collected six biological replicates of 20 berries per replicate at each of five stages (green hard, green soft, red/purple, blue, ripe) from field-grown Merlot, Syrah, and Concord vines. After immediate transport to the laboratory, the pedicels were cutoff from the berries and stored at −80°C until ribonucleic acid (RNA) extraction. The pedicels were ground under liquid nitrogen with a mortar and a pestle, and RNA was isolated and purified as described previously (Reid et al., 2006).

For quantitative reverse transcription–polymerase chain reaction (RT–qPCR) analysis, 2 μg of total RNA were reverse-transcribed with oligo(dT)12–18 in a 20-μL reaction mixture using the SuperScript III (Invitrogen) according to the manufacturer’s instructions. The complimentary deoxyribonucleic acid (cDNA) obtained was diluted 10-fold in ultrapure water. Primer pairs amplifying a *GAPDH* (glyceraldehyde-3-phosphate dehydrogenase) gene and spanning one intron were used to check the absence of genomic DNA contamination with regular PCR. RT-qPCR expression analysis was performed using a commercial iQ SYBR Green Supermix (Bio-Rad), according to the manufacturer’s instructions, with the CFX96 Real-Time PCR Detection system (Bio-Rad). Reaction mixes (10 μL) included 5 μL of iQ SYBR Green Supermix (Bio-Rad), 2 μL of diluted cDNA, and 0.2 μM of each primer. Specificity of primers of all genes of interest (*HT1, HT2, HT3, HT4, HT5, HT6, HT7, SUC11, SUC12*, and *SUC27*; for primer sequences and GenBank accession numbers of these genes, see Supplemental Table S1) was tested and confirmed. Gene transcripts were quantified upon normalization to internal standards of the reference genes *GAPDH* and *Actin*. All values were expressed relative to the value of the least expressed gene. All biological samples were tested in triplicate. Effects of genotypes, genes, and developmental stages were tested by factorial analysis of variance (ANOVA) in Statistica 12 (TIBCO Software). Due to significant interaction among these factors, one-way ANOVA was then performed for each gene within each genotype, and differences during berry development were evaluated using Fisher’s least significant difference test.

### Immunolocalization of sugar transporters

Pedicel sections of green hard, green soft, red/purple, blue, and ripe Merlot, Syrah, and Concord berries were fixed overnight at 4°C in 50-mM Pipes buffer with 1.25% (v/v) glutaraldehyde and 2% (v/v) paraformaldehyde. After three washes with 50-mM Pipes buffer the samples were dehydrated with an increasing sequence of ethanol (30%, 50%, 60%, 70%, 80%, 95% v/v) in water during 10 min each at room temperature and with 100% ethanol for 2 days at 4°C. Then the samples were infiltrated with an increasing sequence in LR White resin:ethanol solution (1:3, 1:2, 1:1, 3:1) overnight and 100% LR White resin for 3 days at 4°C. Finally, samples were embedded in LR White resin in gelatin capsules.

The immunofluorescence reaction was carried out in the dark on sections of 0.5–1 µm thickness from embedded pedicels, using a modified procedure from Fleurat-Lessard et al. (1997). Briefly, sections were placed in Tris-buffered saline with 0.1% (v/v) Triton X-100 (TBS-T) and 0.5% (v/v) glycine for 15 min at room temperature. After three washes in TBS-T, slides were blocked for 2 h at room temperature in TBS-T with 0.2% (v/v) Tween-20, 0.1% (w/v) bovine serum albumin (BSA), and 10% (v/v) goat serum. Then the sections were incubated for 48 h at 4°C with αSUC27 antibody (10 µg mL^−1^ in TBS with 1% w/v BSA; GenScript USA). Controls were prepared by omitting the primary antibody (control 1) or by using the pre-immune serum (1:100 dilution in TBS with 1% BSA) (control 2). After three washes in TBS with 1% BSA, the sections were incubated for 1 h at 4°C with Texas Red conjugate goat anti-Rabbit IgG (10 µg mL^−1^ in TBS with 1% BSA and 0.1% Triton X-100; Life Technologies). After three washes with TBS, auto-fluorescence was reduced by Evans blue (1:10,000 dilution in TBS; Sigma-Aldrich). Sections were mounted with ProLog Diamond antifade with DAPI (Life Technologies). Images were taken using an AxioCam MRm camera on a PALM MicroBeam IV fluorescence microscope (Carl Zeiss Microscopy) with excitation and emission wavelengths of 595 nm and 620 nm, respectively, for the Texas Red antibody and 359 nm and 461 nm, respectively, for DAPI, exposure time of 20 s, and no gain applied.

### Light and transmission electron microscopy

Pedicel segments (∼1–2 mm) of green hard, green soft, and ripe Concord, Merlot, and Syrah berries (three replicates of individual pedicels per developmental stage) were cut in the laboratory immediately after sample collection in the field and fixed overnight at 4°C with 3% (v/v) glutaraldehyde in 0.1-M potassium phosphate buffer at pH 7.2. After three rinses in the phosphate buffer, segments were post-fixed in 2% (w/v) osmium tetroxide for 2 h at room temperature. Subsequently, segments were dehydrated in an ethanol series (30%–100% v/v) and infiltrated with propylene oxide/Spurrs resin prior to embedding. Cross sections were cut at 800 μm on an ultramicrotome (Reichert-Jung), stained with 1% (w/v) Toluidine blue in 1% (v/v) sodium borohydrate for 2 min with heat, and observed under a light microscope (Olympus BH-2) to examine pedicel anatomy. Ultrathin sections (80 μm) were cut with the ultramicrotome, picked up with 200 mesh nickel grids, and stained with uranyl acetate and then Reynolds lead for 8 min each. Observation and imaging of the ultrastructure of pedicel vascular tissues were performed by TEM (FEI Tecnai G^2^ T20, Field Emission Instruments).

### CFDA feeding and analysis

A 20-g L^−1^ stock solution of CFDA (Invitrogen) was prepared in acetone and stored at −20°C. The stock solution was diluted to 1-g L^−1^ working solution (Wright and Oparka, 1996; Zhang et al., 2006) before each experiment. We collected fruit clusters with berries ranging from green hard to ripe from field-grown Concord and Syrah vines, and immediately immersed the cut peduncle in CFDA working solution. Feeding was continued for 24 h after clusters were transferred to the laboratory. We examined only berries ≥10-cm distant from the cut end of the clusters in order to avoid any interference of dye transport in damaged tissues (Botha et al., 2008). Cross sections of pedicels were cut using fresh razor blades under silicone oil to minimize fluorophore loss. The sections were mounted in silicone oil and immediately examined through a fluorescence microscope (Olympus BX51), with 488 nm and 530 nm as excitation and emission wavelengths, respectively. To minimize interference by lignin autofluorescence, we used a wide ultraviolet filter (Hutzler et al., 1998) and controlled exposure rate at ≤70 ms. Green hard, green soft, and ripe berries were examined, and at least three pedicels per genotype were observed at each stage.

### Accession Numbers

Sequence data from this article can be found in the GenBank/EMBL data libraries under the accession numbers listed in Supplemental Table S1.

## Supplementary data

The following materials are available in the online version of this article.

**Supplemental Table S1.** Sequences of primers and the GenBank accession numbers of the genes used in gene expression analysis.

**Supplemental Figure S1.** Pedicel cross section of a green hard Merlot grape berry.

## Supplementary Material

kiac262_Supplementary_Data

## Notes

Y.Z. and M.K. conceived and designed the original study and wrote the article with contributions from all authors; Y.Z., B.-M.C., and B.B. performed the experiments and analyzed the data; Z.D. and S.D. contributed intellectually and provided support to Y.Z. conducting the gene expression analysis; M.K. supervised the study. All approved the final version of the manuscript.

The author responsible for distribution of materials integral to the findings presented in this article in accordance with the policy described in the Instructions for Authors (https://academic.oup.com/plphys/pages/general-instructions) is: Markus Keller (mkeller@wsu.edu)
