# Supplementary material for: Apoplastic sugar may be lost from grape berries and retrieved in pedicels
Source: Plant Physiol. 2022 Jun 1;190(1):592–604. doi: 10.1093/plphys/kiac262 (PMC9434297; doi:10.1093/plphys/kiac262)
Supplement: kiac262_Supplementary_Data [file kiac262_supplementary_data.pdf]

Apoplastic Sugar May be Lost from Grape Berries and Retrieved in Pedicels

Supplemental Table S1. Sequences of primers and the GenBank accession numbers of the genes used in gene expression analysis.

Gene	Primer sequence (5'-3')[a]	Reference	GenBank Accession number
HT1	F: CGTTGTTCACATCGTCGCTTTATC	Lecourieux et al., 2010	AJ001061
	R: GAGCAGTCCTCCGAATAGCATTG		
HT2	F: GGCATAGGGGTGGTGGTAG	Lecourieux et al., 2010	AY663846
	R: TCGGGCTTTACGGAGAGAG		
HT3	F: GCTACTCTCTACTCGTCCGCTTTG	Lecourieux et al., 2010	AY538259
	R: CCCGTCATTGCTTCCGAACTTG		
HT4	F: GGGCTGGCGAGTTTCTCTAG	Lecourieux et al., 2010	AY538260
	R: AGTTCTGCTTGGACATCGTTTG		
HT5	F: CAGGCTGTTCCACTGTTCTTATCG	Lecourieux et al., 2010	AY538261
	R: AGTTAGGAGGACCGCAGGAATC		
HT6	F: TTTATTTGCATGAGGAGGGAGTC	Lecourieux et al., 2010	AY861386
	R: GAGCAGCAGCCTGGATATAATC		
HT7	F: TTACCTCTTCCCTCTACCTTGCTG	Lecourieux et al., unpublished[a]	AY854146
	R: TCGCCAAATCCAATGCCAATACC		
SUC11	F: CTCACGCCTGGTCCAGTATC	Lecourieux et al., unpublished	AF021808
	R: CCGATGTCAGCCGAGAATCC		
SUC12	F: CAAGAATCTGAAGCAGGGTGAG	Lecourieux et al., unpublished	AF021809
	R: GGCGGCAATCATACAACTGAG		
SUC27	F: GGAGTTAGCCAAGCCTTCTTCAG	Lecourieux et al., unpublished	AF021810
	R: GCAACGCCCATCCGAACTG		
Actin	F: CTTGCATCCCTCAGCACCTT	Reid et al., 2006	EC969944
	R: TCCTGTGGACAATGGATGGA		
GAPDH	F: TTCTCGTTGAGGGCTATTCCA	Reid et al., 2006	CB973647
	R: CCACAGACTTCATCGGTGACA		

[a]Specific primer pairs were designed with Beacon Designer 7 software (Premier Biosoft International). Dissociation kinetics at the end of each PCR run were used to verify specific annealing of the oligonucleotides. The efficiency of each primer pair was quantified on a serial dilution of PCR products.

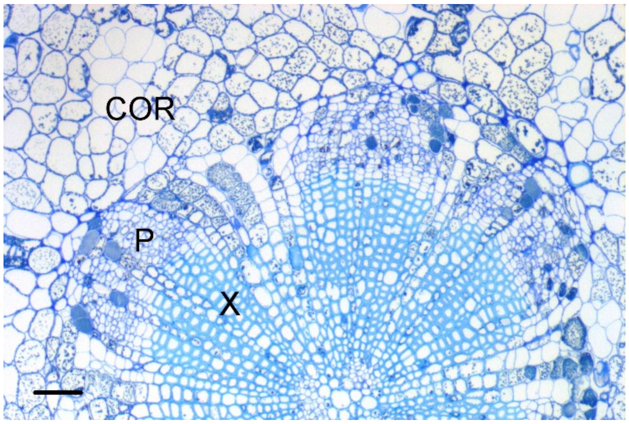

Supplemental Figure S1. Pedicel cross-section of a green hard Merlot grape berry. Samples were cut at 0.8 µm thickness and stained with 1% Toluidine blue in 1% sodium borohydrate for 2 min with heat, and the picture was taken under a light microscope. Abbreviations: COR, cortex; P, phloem; X, xylem. Bar = 100 µm.
